# Patterns and contextual determinants of antibiotic prescribing for febrile under-five outpatients at primary and secondary healthcare facilities in Bugisu, Eastern Uganda

**DOI:** 10.1093/jacamr/dlac091

**Published:** 2022-09-05

**Authors:** Gbemisola Allwell-Brown, Juliet Sanyu Namugambe, Jacquellyn Nambi Ssanyu, Emily White Johansson, Laith Hussain-Alkhateeb, Susanne Strömdahl, Andreas Mårtensson, Freddy Eric Kitutu

**Affiliations:** Department of Women’s and Children’s Health, International Maternal and Child Health (IMCH), Uppsala University, SE-751 85 Uppsala, Sweden; Department of Pharmacy, Faculty of Medicine, Mbarara University of Science and Technology, PO Box 1410, Mbarara, Uganda; Sustainable Pharmaceutical Systems (SPS) Unit, School of Health Sciences, Makerere University, PO Box 7072, Kampala, Uganda; School of Public Health, Makerere University, PO Box 7072, Kampala, Uganda; Department of Women’s and Children’s Health, International Maternal and Child Health (IMCH), Uppsala University, SE-751 85 Uppsala, Sweden; Global Health, School of Public Health and Community Medicine, Institute of Medicine, Sahlgrenska Academy, University of Gothenburg, SE-405 30 Gothenburg, Sweden; Section of Infectious Diseases, Department of Medical Sciences, Uppsala University, SE-751 85 Uppsala, Sweden; Department of Women’s and Children’s Health, International Maternal and Child Health (IMCH), Uppsala University, SE-751 85 Uppsala, Sweden; Department of Women’s and Children’s Health, International Maternal and Child Health (IMCH), Uppsala University, SE-751 85 Uppsala, Sweden; Sustainable Pharmaceutical Systems (SPS) Unit, School of Health Sciences, Makerere University, PO Box 7072, Kampala, Uganda; School of Public Health, Makerere University, PO Box 7072, Kampala, Uganda

## Abstract

**Objectives:**

To describe patterns and contextual determinants of antibiotic prescribing for febrile under-five outpatients at primary and secondary healthcare facilities across Bugisu, Eastern Uganda.

**Methods:**

We surveyed 37 public and private-not-for-profit healthcare facilities and conducted a retrospective review of antimicrobial prescribing patterns among febrile under-five outpatients (with a focus on antibiotics) in 2019–20, based on outpatient registers. Multilevel logistic regression analysis was used to identify determinants of antibiotic prescribing at patient- and healthcare facility-levels.

**Results:**

Antibiotics were prescribed for 62.2% of 3471 febrile under-five outpatients. There were a total of 2478 antibiotic prescriptions of 22 antibiotic types: amoxicillin (52.2%), co-trimoxazole (14.7%), metronidazole (6.9%), gentamicin (5.7%), ceftriaxone (5.3%), ampicillin/cloxacillin (3.6%), penicillin (3.1%), and others (8.6%). Acute upper respiratory tract infection (AURTI) was the commonest single indication for antibiotic prescribing, with 76.3% of children having AURTI as their only documented diagnosis receiving antibiotic prescriptions. Only 9.2% of children aged 2–59 months with non-severe pneumonia received antibiotic prescriptions in line with national guidelines. Higher health centre levels, and private-not-for-profit ownership (adjusted OR, 4.30; 95% CI, 1.91–9.72) were significant contextual determinants of antibiotic prescribing.

**Conclusions:**

We demonstrated a high antibiotic prescribing prevalence among febrile under-five outpatients in Bugisu, Eastern Uganda, including prescriptions for co-trimoxazole and ampicillin/cloxacillin (which are not indicated in the management of the common causes of under-five febrile illness in Uganda). Study findings may be linked to limited diagnostic capacity and inadequate antibiotic availability, which require prioritization in interventions aimed at improving rational antibiotic prescribing among febrile under-five outpatients.

## Introduction

Antimicrobial resistance (AMR) is a global challenge, with worse health and economic implications for low- and middle-income countries (LMICs).^1^ Uganda, an East-African low-income country, adopted a National Action Plan (NAP) on AMR in 2018,^2^ but as with other African LMICs, implementation has been slow.^3^ Promoting the optimal use of antimicrobials is a key objective of Uganda’s NAP, but inadequate local data on current patterns and determinants of antimicrobial prescribing and use hampers the implementation of context-relevant antimicrobial stewardship interventions at health facilities.^2^

Children under 5 years old are an important group of antibiotic users, one for which antibiotics are often over-prescribed and inappropriately prescribed.^4,5^ Importantly, the commonest conditions in the under-five age group for which antibiotics are most often inappropriately prescribed [such as acute upper respiratory tract infections (AURTI) and acute watery diarrhoea]^5–8^ are largely managed on an outpatient basis at primary and secondary healthcare facilities. Hence, understanding antibiotic prescribing practices especially at these levels of healthcare is a prerequisite for optimizing antimicrobial use and improving health outcomes for young children. However, evidence on antibiotic prescribing patterns among paediatric outpatients in primary and secondary healthcare facilities in Uganda remains scarce.^9^

Prescribing behaviour is complex, and is influenced not only by the patient–provider interaction, but also by broader healthcare facility and health system factors.^10^ In Uganda and similar settings, contextual health system factors such as heavy workload for healthcare workers, and limited availability of essential diagnostics and antibiotics may contribute to inappropriate antibiotic prescribing.^11,12^ Yet, to our knowledge, little is known about the influence of contextual health system factors on antibiotic prescribing for paediatric outpatients in Uganda. Such evidence could further elucidate modifiable drivers of antibiotic prescribing, which is important for the design of context-appropriate, sustainable antimicrobial stewardship interventions in Ugandan outpatient settings and similar contexts elsewhere. Therefore, this study aimed to describe patterns and contextual determinants of antibiotic prescribing for febrile under-five outpatients at primary and secondary healthcare facilities in Bugisu, Eastern Uganda.

## Patients and methods

### Study design and study setting

This was a cross-sectional study conducted in outpatient settings of 37 primary and secondary healthcare facilities across the six districts of Bugisu (Bulambuli, Sironko, Bududa, Manafwa, Mbale and Namisindwa). Bugisu, a sub-region in Eastern Uganda, is a malaria-endemic setting, with an estimated population of 1.8 million inhabitants,^13^ and has under-five mortality and poverty rates higher than the national average.^14,15^

Healthcare in Uganda is based on a referral system and a tiered healthcare structure, and is delivered through public and private healthcare facilities. Formal healthcare facilities in Uganda are organized into six levels, according to the services provided and catchment area covered, as described elsewhere.^16^ Health Centre level two (HC-II) and HC-III are primary healthcare centres; HC-IV and general hospitals provide secondary healthcare services; and tertiary-level healthcare services are provided at regional- and national referral hospitals.^16,17^ The lead role in prescribing is taken by nurses at HC-II, Clinical Officers at HC-III, and Medical Doctors, where available, at higher healthcare levels. Clinical Officers are non-physician clinicians with training in clinical aspects of medicine, and with fewer clinical skills than physicians but more than basic nurses.^18^

This study included public and private-not-for-profit healthcare facilities at four levels: HC-II, -III, -IV, and general hospital. There were two study components: a healthcare facility survey, and a register-based retrospective review of antimicrobial prescribing patterns among febrile under-five outpatients [children aged under 5 years (including neonates) with febrile illness that were managed as outpatients] at the study healthcare facilities, from January 2019 to December 2020.

### Selection of healthcare facilities

Multi-stage and purposive sampling were used in the selection of healthcare facilities for this study, to ensure representation of the six districts, and the multiple levels of healthcare provision in Bugisu. First, two general hospitals and 10 HC-IV facilities in Bugisu were enrolled. Second, 16 HC-III and 11 HC-II were purposively selected, taking into account representation of the districts, health sub-districts, and ownership (public versus private-not-for-profit). One general hospital was excluded because it provided only specialized services, which did not match the aim of the study, and one HC-IV was excluded due to unavailability of the medical records staff on all occasions the facility was visited for data collection. Altogether, 37 healthcare facilities were enrolled, six of which were private-not-for-profit. (see Table S1, available as Supplementary data at *JAC-AMR* Online)

### Sampling of febrile under-five outpatients

Records of febrile under-five outpatients that presented to the study healthcare facilities from 1 January 2019 to 31 December 2020 were considered eligible for inclusion. Records were excluded if: (i) fever was not documented as a presenting clinical sign or symptom, or (ii) records had incomplete or illegible entries about the diagnoses and prescriptions.

Records were selected systematically at the health facilities. To obtain the sampling interval, the total number of under-five children attending the outpatient clinics at each of the facilities within the study period was first obtained. This was determined using health facility monthly reports from the Health Information Management System Form 105,^19^ where these were available, or by tallying daily attendances from the outpatient registers. This was then divided by the required sample size at that healthcare facility to obtain a sampling interval. Records were then selected by the determined sampling interval, taking the first eligible child attending in January 2019 as the starting point. Whenever an ineligible record was encountered, it was skipped and replaced with the next eligible record without breaking the original sampling interval.

### Study size for febrile under-five outpatients

Sample size was estimated using the Kish Leslie formula for cross-sectional studies,^20^ using a 5% level of precision and a 95% CI. The proportion of under-five children prescribed an antibiotic was assumed as 84.9%, based on a similar study conducted among paediatric inpatient and outpatients in Tanzania.^21^ This yielded a sample size of 196, which was adjusted to cater for clustering at the four levels of service delivery (HC-II, -III, -IV and General Hospital), with a 10% allowance for missing data and incomplete records, resulting in an estimated sample size of 2309 (for details see Supplementary data).

### Data sources

Data on healthcare facility characteristics were collected via a pre-tested, structured questionnaire administered to the persons-in-charge at each facility, combined with inspection, using a checklist. (Supplementary data). Data were collected about healthcare facility level and ownership, services provided, diagnostics availability, patient attendance at outpatient clinics, staffing, and availability of Uganda Clinical Guidelines (UCG).

For the retrospective assessment of prescribing patterns for febrile under-five outpatients, data from outpatient registers were collected using an electronic data abstraction tool developed by the investigators (Supplementary data). Outpatient registers are physical documents used for recording details of patients attending the outpatient departments of healthcare facilities in Uganda. They capture patient data including their name, age, sex, weight, height, residence, body temperature, diagnostic tests performed, diagnosis, medicines prescribed (including dosage), attendance classification (new- or re-attendance), and referral details.

### Assessment of antimicrobial prescribing patterns for febrile under-five outpatients

Each medication prescribed was considered as one prescription, such that one child could have multiple prescriptions. All prescriptions were assessed to determine the proportion of antimicrobial agents in any form (topical or systemic), which were then classified as antibacterials, antimalarials, antifungals, or antiviral agents. Antibiotics were defined as antibacterial medications belonging to the Anatomical Therapeutic Chemical (ATC) J01 (Antibacterials for systemic use), and P01AB (Nitroimidazole derivatives) classes,^22,23^ and further categorized by WHO Access, Watch and Reserve (AWaRe) group.^24,25^ Access antibiotics have the lowest resistance-development potential, and should be widely accessible as first-line antibiotics for most indications; Watch antibiotics are mainly used as second-line treatment; and Reserve antibiotics with the greatest resistance-development potential are last-resort medications.^24,25^ Where feasible, prescriptions and their corresponding indications were compared with the UCG,^26^ to assess degree of congruence. Descriptive statistics were summarized using a bar graph, pie chart and cross-tabulations.

### Variables and statistical analyses

Multilevel logistic regression was used to investigate the determinants of antibiotic prescribing at patient- (Level-1) and healthcare facility-levels (Level-2), taking the 37 healthcare facilities as clusters. The outcome variable was dichotomous, indicating whether a child had a documented prescription for any oral or parenteral antibiotic (ATC J01/P01AB) or not. Level-1 variables included in the models were: child’s age (<6 months, 6–24 months, or >24 months), child’s sex, date of presentation (obtained by dividing the 2 year study period into successive 3 month periods, to account for seasonality), child’s malaria test result, and dichotomous variables for the presence or absence of the commonest reported diagnoses. At Level-2, the following variables were included: health centre level (HC-II, HC-III, HC-IV, or General Hospital), healthcare facility ownership (public or private-not-for-profit), availability of UCG, and a continuous variable representing patient-to-prescriber ratio, obtained by dividing the average outpatient attendance over the 5 working days before the survey, by the total number of prescribers available at the healthcare facility. (At HC-II and HC-III, nurses and Clinical Officers were considered as prescribers, and at HC-IV and General Hospital, Clinical Officers and Medical Doctors were considered as prescribers).

The following models were run: Model 1, the null-model containing only the outcome variable and the healthcare facility identifier (cluster) variable; Model 2, which included Level-1 variables with the facility identifier; and Model 3, which included Level-1 and Level-2 variables with the facility identifier. The level of statistical significance was set at 0.05. Variance inflation factor (VIF) was used to assess covariates for multicollinearity. The mean VIF was 1.14 (multicollinearity was defined as a VIF value of 10 or greater). Data were analysed using Stata 15.1 (Stata Corp., College Station, TX).

### Ethics

Ethics approval was obtained from the Makerere University School of Health Sciences Research and Ethics Committee, reference number MakSHSREC-2020-21. The study was registered and cleared by the Uganda National Council for Science and Technology, reference number HS1155ES.

## Results

### Characteristics of surveyed healthcare facilities and febrile under-five outpatients

Characteristics of the 37 surveyed healthcare facilities (11 HC-II, 16 HC-III, 9 HC-IV and 1 General Hospital) are shown in Table 1. Malaria rapid diagnostic test (mRDT) was the most widely available diagnostic tool (available in 36/37 healthcare facilities), and UCG were available in 32 healthcare facilities.

**Table 1.** Characteristics of surveyed primary and secondary healthcare facilities in Bugisu, Eastern Uganda

Healthcare facility characteristics	HC-II (*n *= 11)	HC-III (*n *= 16)	HC-IV (*n *= 9)	General Hospital (*n *= 1)	Total (*N *= 37)
**Ownership**					
Public	9	14	7	1	31
Private-not-for-profit	2	2	2	0	6
**Average no. of staff per healthcare facility**					
Doctors	0	0	2.7	5	-
Clinical officers	0.1	1.4	3.6	4	-
Nurses and midwives	3.3	6.2	16.9	59	-
Pharmacists	0	0	0	1	-
Dispensers	0	0.06	0.3	2	-
Laboratory personnel	0.5	1.3	3.1	8	-
Records personnel	0.2	0.9	1.7	2	-
**Average outpatient clinic attendance over 5 working days preceding the survey**	16.8	27.6	71.4	104.4	-
**Diagnostics availability**					
Malaria RDT	10	16	9	1	36
Malaria microscopy	3	13	9	1	26
Complete blood count	0	2	1	1	4
Typhoid test	3	1	4	1	9
Urinalysis	2	14	7	1	24
Gram stain	1	8	6	0	15
Culture and sensitivity	0	0	0	0	0
Drug susceptibility test	0	0	0	0	0
Tuberculosis test	1	12	5	1	19
HIV test	9	16	7	1	33
COVID-19 test	1	0	2	0	3
**Availability of Uganda Clinical Guidelines**	10	13	8	1	32

Records of 3598 febrile under-five outpatients that attended the surveyed healthcare facilities between 1 January 2019 and 31 December 2020 were retrospectively reviewed. The majority, 3583 (99.6%), were reported as first-time presentations, 1774 (49.3%) were male; 1883 (52.3%) were aged 6–23 months, (Table 2), with 132 (3.6%) aged 2 months or younger (including three neonates).

**Table 2.** Background and clinical characteristics of surveyed febrile under-five outpatients attending lower-level healthcare facilities in Bugisu, Eastern Uganda in 2019 and 2020

		Children receiving at least one ATC J01/P01AB antibiotic, *n* (%)		
Variable	*n* (%) (*N *= 3598)	No (%)	Yes (%)	Not reported (%)
**Child’s age**				
<6 months	349 (9.7%)	88 (25.2%)	246 (70.5%)	15 (4.3%)
6–23 months	1883 (52.3%)	668 (35.5%)	1150 (61.1%)	65 (3.5%)
≥24 months	1366 (38.0%)	557 (40.8%)	762 (55.8%)	47 (3.4%)
**Child’s sex**				
Male	1774 (49.3%)	637 (35.9%)	1068 (60.2%)	69 (3.9%)
Female	1824 (50.7%)	676 (37.1%)	1090 (59.8%)	58 (3.2%)
**Year of attendance**				
2019	1838 (51.1%)	688 (37.4%)	1073 (58.4%)	77 (4.2%)
2020	1760 (48.9%)	625 (35.5%)	1085 (61.7%)	50 (2.8%)
**Attendance**				
New attendance	3583 (99.6%)	1309 (36.5%)	2148 (60.0%)	126 (3.5%)
Re-attendance	15 (0.4%)	4 (26.7%)	10 (66.7%)	1 (6.7%)
**Diagnostic test**				
Malaria RDT	2415^a^ (67.1%)	1001 (41.4%)	1333 (55.2%)	81 (3.4%)
Malaria microscopy	344^a^ (9.6%)	119 (34.6%)	208 (60.5%)	17 (4.9%)
Gram staining	1 (0.03%)	0 (0.0%)	1 (100.0%)	0 (0.0%)
Not reported	838 (23.3%)	193 (23.0%)	616 (73.5%)	29 (3.5%)
**Diagnosis** ^b^				
Malaria	1414 (39.3%)	577/680^c^ (84.9%)	103/680^c^ (15.2%)	-
AURTI	1381 (38.4%)	160/676^d^ (23.7%)	516/676^d^ (76.3%)	-
Diarrhoea^e^	548 (15.2%)	115/192^f^ (59.9%)	77/192^f^ (40.1%)	-
Pneumonia^g^	370 (10.3%)	12/356^h^ (3.4%)	344/356^h^ (96.6%)	-
Skin infections	227 (6.3%)	23/120^i^ (19.2%)	97/120^i^ (80.8%)	-
Non-specific bacterial infections^j^	200 (5.6%)	5/143^k^ (3.5%)	138/143^k^ (96.5%)	-
Helminthiasis	154 (4.3%)	9/15^l^ (60.0%)	6/15^l^ (40.0%)	-

a Malaria RDT and microscopy were reported in four cases. These are grouped as RDT.

b Categories are not exclusive, and only the most common diagnoses (reported in at least 100 cases) are listed.

c Denominator is 680 cases with malaria as the only recorded diagnosis, supported by documented positive malaria test.

d Denominator is 676 cases with AURTI as the only recorded diagnosis.

e Includes 526 cases of acute watery diarrhoea, 14 cases of dysentery and 8 cases of persistent diarrhoea.

f Denominator is 192 cases with acute watery diarrhoea as the only recorded diagnosis.

g Includes 337 cases of non-severe pneumonia and 33 cases of severe pneumonia.

h Denominator is 356 cases with a diagnosis of pneumonia (severe or non-severe), regardless of any other diagnosis.

i Denominator is 120 cases with skin infection as the only recorded diagnosis.

j Includes the diagnoses ‘bacteraemia’, ‘septicaemia’, ‘sepsis’, and ‘bacterial infection’.

k Denominator is 143 cases with non-specific bacterial infection as the only recorded diagnosis.

l Denominator is 15 cases with helminthiasis as the only recorded diagnosis.

### Antimicrobial prescribing patterns

The majority, 3471 (96.8%), of the 3598 surveyed febrile under-five outpatients were prescribed at least one medication, resulting in a total of 9745 prescriptions. On average, each febrile under-five outpatient received 2.81 prescriptions per clinical encounter, with 487 (14.0%) receiving at least one injection prescription.

**Figure 1.**
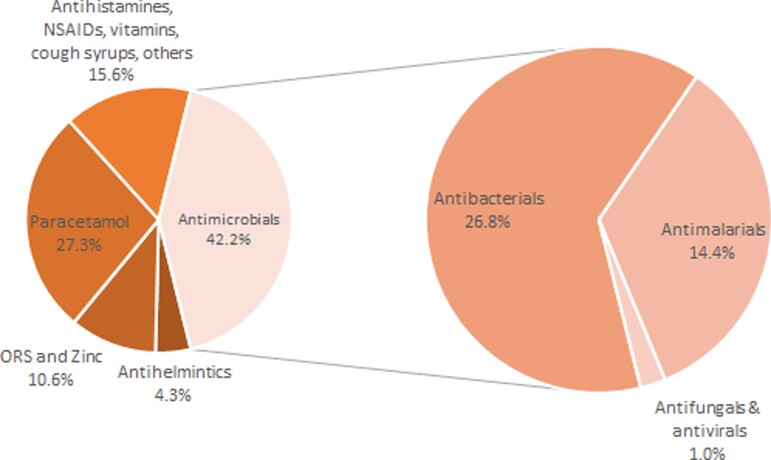
Distribution of all prescriptions for febrile under-five children attending study healthcare facilities in Bugisu, Eastern Uganda in 2019 and 2020 (*N *= 9745).

Of the total 9745 prescriptions, 4114 (42.2%) were for antimicrobials in any form (Figure 1). Of these, 2611 (63.5%) were antibacterials, 1407 (34.2%) were antimalarials, 95 (2.3%) were antifungals, and 2 (0.05%) were antivirals; comprising 26.8%, 14.4%, 0.9% and 0.02% of all prescriptions, respectively (Figure 1). Among antibacterial prescriptions, 94.5% were for systemic administration in the form of injectable or oral formulations (ATC J01/P01AB), while the remaining were topical (creams, ointments and eye-drops).

**Figure 2.**
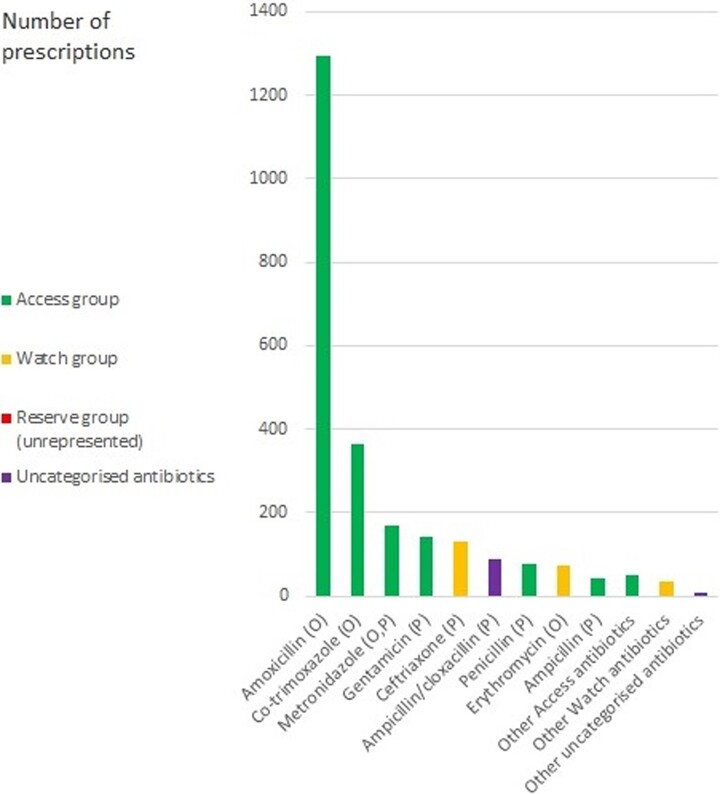
Frequency of antibiotic prescriptions for febrile children under-five attending lower-level healthcare facilities in Bugisu, Eastern Uganda in 2019 and 2020 by AWaRe classification. O, oral preparation; P, parenteral preparation. A list of the ‘Other’ antibiotics and their frequency of prescription is available in the Supplementary data.

A total of 22 different antibiotics were prescribed, including 12 and 6 from the WHO Access and Watch categories, respectively, and 4 unclassified antibiotics (Figure 2). There were no prescriptions of Reserve group antibiotics documented. Altogether, there were 2478 antibiotic prescriptions. Topmost among these were prescriptions for amoxicillin (52.2%), co-trimoxazole (14.7%), metronidazole (6.9%), gentamicin (5.7%), from the Access group; ceftriaxone (5.3%) from the Watch-group; and ampicillin/cloxacillin (3.6%) from the unclassified group (Figure 2).

### Clinical characteristics and antimicrobial prescribing for febrile under-five outpatients

The commonest diagnoses reported were malaria [1414 (39.3%)], AURTI [1381 (38.4%)], diarrhoea [548 (15.2%)], and pneumonia [370 (10.3%)]. Other diagnoses included skin infections in 227 (6.3%), non-specific bacterial infections (‘bacteraemia’, ‘septicaemia’, ‘sepsis’, and ‘bacterial infection’) in 200 (5.6%), and helminthiasis in 154 (4.3%) children. Diagnostic tests were reportedly performed in 2760 (76.7%) of the 3598 surveyed children (Table 2). The diagnostic tests performed were almost exclusively malaria tests, with only one case where Gram staining was reported (Table 2). Of the 2759 children that had malaria test results available, 1344 (48.7%) were malaria positive either by mRDT or microscopy.

Of 3471 children with documented prescriptions, 3044 (87.7%) received at least one antimicrobial: 2158 (62.2%) received a prescription of at least one antibiotic, 1327 (38.2%) at least one antimalarial, 95 (2.7%) an antifungal, and 2 (0.06%) an antiviral. 484 (13.9%) received both an antibiotic and an antimalarial. The number of antibiotics prescribed per patient ranged from 0 to 4, with patients receiving on average 0.74 antibiotics prescribed per clinical encounter.

### Antibiotic prescribing for AURTI, pneumonia, malaria, and diarrhoea

AURTI was the commonest single indication for antibiotic prescribing. Of 676 children with AURTI as the only documented diagnosis, 76.3% received at least one antibiotic prescription (Table 2). At least 592 (45.7%) amoxicillin prescriptions and 174 (47.8%) co-trimoxazole prescriptions were for AURTI (after exclusion of possible prescribing for co-existing pneumonia, dysentery, otitis media, or urinary tract infections).

Of 314 children aged 2–59 months diagnosed with non-severe pneumonia, 303 (96.5%) received at least one antibiotic prescription, with 226 (72.0%) receiving prescriptions for amoxicillin. Only 29 children (9.2%) received a prescription for amoxicillin at the appropriate dose for up to 5 days, in accordance with national guidelines.^26^ Irrespective of age, amoxicillin for non-severe pneumonia among children aged 2–59 months was mostly prescribed at 250 mg three times daily [104/221 (47.0%)], and for 5 days [184/218 (84.4%)].

Of 680 children with malaria as the only documented diagnosis (supported by a positive malaria test), 103 (15.2%) received at least one antibiotic prescription. Similarly, out of 192 children with a single diagnosis of acute watery diarrhoea, 77 (40.1%) received at least one antibiotic prescription (Table 2). All 13 children with dysentery, and all 30 children with severe pneumonia diagnosis received at least one antibiotic, though choice and combinations of antibiotics varied, and in almost all cases did not match guideline recommendations.

### Determinants of antibiotic prescribing

After adjusting for Level-1 variables, 22.0% of the variation in antibiotic prescribing for febrile under-five outpatients was attributed to between-healthcare facility differences. (Model 2, Table 3). In the final model, 11.4% of this variation remained unexplained (Model 3, Table 3). Level-1 variables that significantly increased the odds of antibiotic prescribing were: negative malaria test result [adjusted odds ratio (AOR) 4.80; 95% CI 3.74–6.16], a diagnosis of pneumonia, AURTI, skin infection, or non-specific bacterial infection (Model 3, Table 3). On the other hand, a diagnosis of helminthiasis [AOR (95% CI) 0.61 (0.37–0.99)], and visiting a healthcare facility in July–September, 2020 [AOR (95% CI) 0.56 (0.36–0.86)] significantly lowered the odds of antibiotic prescribing (Model 3, Table 3).

**Table 3.** Patient- and healthcare facility-level determinants of ATC J01/P01AB antibiotic prescribing for febrile outpatients under-five at surveyed healthcare facilities in Bugisu, Eastern Uganda

	AOR (95% CI)^a^		
Characteristic	Model 1	Model 2	Model 3
**Child’s age**			
<6 months	-	Ref	Ref
6–24 months	-	1.04 (0.69–1.56)	1.04 (0.69–1.57)
>24 months	-	1.07 (0.70–1.64)	1.09 (0.71–1.68)
**Child’s sex**			
Male	-	Ref	Ref
Female	-	0.97 (0.79–1.19)	0.97 (0.79–1.19)
**Date of presentation**			
Jan-Mar 2019	-	Ref	Ref
Apr-Jun 2019	-	0.94 (0.63–1.39)	0.91 (0.61–1.36)
Jul-Sep 2019	-	0.90 (0.60–1.35)	0.90 (0.60–1.35)
Oct-Dec 2019	-	0.70 (0.45–1.10)	0.70 (0.44–1.10)
Jan-Mar 2020	-	0.87 (0.58–1.29)	0.85 (0.57–1.27)
Apr-Jun 2020	-	0.94 (0.61–1.44)	0.95 (0.61–1.46)
Jul-Sep 2020	-	0.55 (0.36–0.84)*	0.56 (0.36–0.86)*
Oct-Dec 2020	-	0.82 (0.54–1.23)	0.81 (0.54–1.22)
**Malaria test result**			
Positive	-	Ref	Ref
Negative	-	4.88 (3.80–6.26)*	4.80 (3.74–6.16)*
**Pneumonia** ^b^			
No	-	Ref	Ref
Yes	-	42.99 (21.86–84.54)*	44.80 (22.01–91.22)*
**AURTI**			
No	-	Ref	Ref
Yes	-	4.96 (3.92–6.27)*	5.18 (4.09–6.57)*
**Acute watery diarrhoea**			
No	-	Ref	Ref
Yes	-	0.71 (0.53–0.96)*	0.75 (0.56–1.01)
**Skin infection**			
No	-	Ref	Ref
Yes	-	5.00 (2.75–9.12)*	5.11 (2.78–9.41)*
**Non-specific bacterial infection** ^c^			
No	-	Ref	Ref
Yes	-	22.62 (9.45–54.10)*	22.42 (9.39–53.56)*
**Helminthiasis**			
No	-	Ref	Ref
Yes	-	0.60 (0.37–0.99)*	0.61 (0.37–0.99)*
**Health centre level**			
HC II	-	-	Ref
HC III	-	-	4.56 (2.43–8.55)*
HC IV	-	-	2.44 (1.23–4.82)*
General hospital	-	-	6.82 (1.42–32.82)*
**Healthcare facility ownership**			
Public	-	-	Ref
Private not for profit	-	-	4.30 (1.91–9.72)*
**Uganda Clinical Guidelines available**			
No	-	-	Ref
Yes	-	-	0.59 (0.27–1.29)
**Patient/prescriber ratio**	-	-	1.04 (1.00–1.09)
**Model measures of clustering**			
Healthcare facility-level variance (standard error)	0.931 (0.248)	0.929 (0.266)	0.425 (0.140)
Proportional change in variance	-	0.21%	54.25%
Intra-class correlation	0.221	0.220	0.114
Median odds ratio	2.51	2.51	1.86

Model 1 is the null model; Model 2 includes Level-1 variables; Model 3 includes Level-1 and Level-2 variables.

a The asterisks indicate results that are statistically significant.

b Includes 337 cases of non-severe pneumonia and 33 cases of severe pneumonia.

c The designation ‘Non-specific bacterial infection’ refers to the following diagnoses: ‘bacteraemia’, ‘septicaemia’, ‘sepsis’, and ‘bacterial infection’.

At Level-2, there were higher odds of antibiotic prescribing at HC-III, HC-IV and General Hospital, compared with HC-II facilities (Model 3, Table 3). Similarly, the odds of antibiotic prescribing were greater in private-not-for-profit healthcare facilities, compared with public facilities [AOR (95% CI) 4.30 (1.91–9.72)] (Model 3, Table 3). A higher patient-to-prescriber ratio and presence of UCG at healthcare facilities showed no significant association with antibiotic prescribing (Model 3, Table 3).

## Discussion

Antibiotics were prescribed to a large proportion (62.2%) of febrile under-five outpatients in this study, with amoxicillin and co-trimoxazole accounting for two-thirds of all antibiotic prescriptions. AURTI was the commonest single indication for antibiotic prescribing, with a prescribing proportion of 76.3% when AURTI was the only documented diagnosis. Notably, only 9.2% of children aged 2–59 months with non-severe pneumonia received an appropriate antibiotic prescription per the national guidelines. Higher health centre levels (compared with HC-II) and private-not-for-profit ownership were significant contextual determinants of antibiotic prescribing.

Antibiotic prescribing proportions of 60.1% and 62.7% have been reported on average for under-five outpatients across LMICs, including Uganda.^5,27^ Given the relatively low prevalence of diagnoses necessitating antibiotic prescribing in this study [pneumonia (10.3%) and dysentery (0.39%)], the overall antibiotic prescribing proportion of 62.2% likely indicates over-prescribing. Akin to reports from other LMICs,^5,6,27–29^ we found antibiotic prescriptions for AURTI, acute watery diarrhoea and malaria, which to a large extent may be unnecessary.^26^ Antibiotic prescribing for other commonly reported conditions, such as skin infections and non-specific bacterial infections was also significant but were less straightforward to appraise. Such generic diagnoses are likely a result of diagnostic uncertainty, considering the limited diagnostic capacity of the study healthcare facilities. Yet, assuming all patients in our study with reported pneumonia, dysentery, skin infections and non-specific bacterial infections required antibiotics for each condition separately, the total prevalence of these conditions (22.6%) would still be well-below the antibiotic prescribing proportion of 62.2%.

Diagnostic test availability was uneven within and between healthcare facility levels, with mRDTs being most commonly available. Similar to reports from other studies,^30^ negative malaria tests significantly increased the odds of antibiotic prescribing. Limited diagnostic capacity for non-malarial fevers remains a major driver of inappropriate antibiotic prescribing in malaria-endemic settings such as Uganda.^31^ Thus, improving the differential diagnostic capacity with microbiology and/or biomarker point-of-care tests for non-malarial fevers at these levels of care could contribute to a reduction in inappropriate antibiotic prescribing.

The antibiotic prescribing patterns observed in this study may be more linked to availability than appropriate indication. For example, amoxicillin and co-trimoxazole, the most commonly prescribed antibiotics in the current study were also the top two antibiotics supplied across public secondary and tertiary healthcare facilities nationwide in 2019 by Uganda’s National Medical Stores (NMS).^17^ And, while amoxicillin is the first-line treatment recommended for most uncomplicated bacterial infections in Uganda, co-trimoxazole is not indicated in the management of any of the conditions reported in this study (its main indication among under-five children in the UCG is as prophylaxis against opportunistic infections in children with HIV).^26^ Yet, co-trimoxazole was the second-most-prescribed antibiotic, accounting for 14.7% of all antibiotic prescriptions. Similarly, ampicillin/cloxacillin, the fifth most commonly distributed antibiotic by the NMS in 2019,^17^ was the most commonly prescribed uncategorized antibiotic in this study, despite having no indication in the national guidelines. The prevalent use of co-trimoxazole and ampicillin/cloxacillin despite their lack of indication is likely a pointer to the inadequate access to effective antibiotics in East-Africa that has been characterized by other authors.^32^

Almost all (96.5%) of the febrile under-five outpatients aged 2–59 months with non-severe pneumonia received an antibiotic prescription. Yet, only 72.0% were prescribed the correct antibiotic (amoxicillin), and more so, only 9.2% received an amoxicillin prescription at the correct dose for the appropriate duration as indicated in the national guidelines. Similarly, although all children with diagnoses of severe pneumonia and dysentery (conditions which necessitate antibiotic treatment) received antibiotics, the choice and combinations of antibiotics prescribed varied—in most cases not in accordance with guidelines. These findings indicate that beyond promoting adequate access to antibiotics where indicated, additional efforts are needed to encourage adherence to national guidelines to ensure their appropriate prescribing.

We found that 22% of the variation in antibiotic prescribing was attributed to determinants at healthcare facility level, after adjusting for patient-level variables. Our results support findings from qualitative studies indicating that, beyond patient and prescriber characteristics, prescribing behaviour is often shaped by contextual factors.^10^ We identified that health centre level and ownership were important contextual determinants of antibiotic prescribing. Our finding that antibiotic prescribing was significantly greater at all levels above HC-II is reasonable. HC-II facilities receive fewer antibiotic supplies, since they generally manage less-complicated cases than the higher-level facilities, most of which would not require antibiotics. Finally, in line with our findings, other studies have also shown higher antibiotic prescribing in private facilities,^33^ where revenue is often directly related to patient volume and medicine sales.

### Limitations

First, this study relied on outpatient registers for data on treatment practices for febrile under-five outpatients. Thus, the study was limited to collected data, and could have been compromised by incomplete or missing data. However, our analysis did not expose any systematic bias attributable to records that were not available at data collection. Second, important data on prescriptions such as dose, interval and duration that were not captured in full for some of the cases made it impossible to determine whether or not patients were prescribed the correct regimen for some of the diagnoses. Third, our determination of patient-to-prescriber ratio was not based on a direct measure of patient load per-prescriber, but represented average patient load at the healthcare facilities and thus could have dampened the effect of this determinant. Future studies could use more direct measures of patient load per shift for each prescriber. Finally, while the analytical statistics took into account the survey and cluster structure of the data by using the mixed-effect approach, the descriptive statistics did not apply weights to account for unequal probabilities of selection due to different client volumes at sampled facilities. Despite the large sample and high response rate, which should reduce the selection bias, we have taken extra caution when interpreting the descriptive findings from this study.

### Conclusions

We demonstrate a high antibiotic prescribing prevalence among febrile under-five outpatients attending primary and secondary healthcare facilities in Bugisu, Eastern Uganda, including the prescribing of co-trimoxazole and ampicillin/cloxacillin, which are not indicated in the management of the common causes of febrile illness in under-five children in Uganda. These prescribing patterns may be linked to limited diagnostic capacity and antibiotic availability at study facilities. Our study results also provide quantitative evidence that health centre level and ownership are significant contextual determinants of antibiotic prescribing for children in Uganda. There is a need to prioritize access to effective antibiotics and essential diagnostics in interventions aimed at optimizing antibiotic use among febrile under-five outpatients. Future interventions will benefit from qualitative research into coping strategies adopted by care-seekers and healthcare workers in such highly resource-constrained settings.

## Supplementary Material

dlac091_Supplementary_Data

## Supplementary data

Additional Methods information, checklists and Table S1 are available as Supplementary data at *JAC-AMR* Online.
