# Supplementary material for: Patterns and contextual determinants of antibiotic prescribing for febrile under-five outpatients at primary and secondary healthcare facilities in Bugisu, Eastern Uganda
Source: JAC Antimicrob Resist. 2022 Sep 5;4(5):dlac091. doi: 10.1093/jacamr/dlac091 (PMC9444054; doi:10.1093/jacamr/dlac091)
Supplement: dlac091_Supplementary_Data [file dlac091_supplementary_data.docx]

**Supplementary data**

**Patterns and contextual determinants of antibiotic prescribing for febrile under-five outpatients at primary and secondary healthcare facilities in Bugisu, Eastern Uganda**

**Authors**

Gbemisola Allwell-Brown, MD (corresponding author)

Juliet Sanyu Namugambe, MSc

Jacquellyn Nambi Ssanyu, MPH

Emily White Johansson, PhD

Laith Hussain-Alkhateeb, PhD

Susanne Strömdahl, PhD

Andreas Mårtensson, MD

Freddy Eric Kitutu, PhD

**Table S1**: Distribution of surveyed healthcare facilities by district, sub-district, level of care, and ownership

| District | Sub-district | HC-II | HC-III | HC-IV | **Total** |
| --- | --- | --- | --- | --- | --- |
| Bududa | Lutseshe | 1 | 1 | - | 2 |
|  | Manjiya | 1 | 1 | - | 2 |
| Bulambuli | Bulambuli | 3 | 4 | 1 | 8 |
| Manafwa | Bubulo West | 2 | 1 | 2 | 5 |
| Mbale | Bungokho North | 1 | 1 | 1 | 3 |
|  | Bungokho South | 1 | 1 | 1 | 3 |
|  | Mbale Municipality | 1 | 3 | 1 | 5 |
| Namisindwa | Bubulo East | 1 | 1 | 1 | 3 |
| Sironko | Budadiri East | - | 2 | 1 | 3 |
|  | Budadiri West | - | 1 | 1 | 2 |
| **Total** |  | 11 | 16 | 9 | **37**^a^ |

*^a^ Total includes one General Hospital which was also surveyed but not otherwise shown in the table. Of the 37 healthcare facilities, six were private and not for profit (PNFP).*

**Sample size calculation**

Sample size was estimated using the Kish Leslie formula for sample size determination for cross-sectional studies^1^ using a 5% level of precision and a 95% confidence interval. The proportion of children under five years prescribed an antibiotic was taken as 84.9% from a cross sectional study conducted in Tanzania assessing antibiotic prescribing patterns in the management of diarrhoea and cough among children under five years old attending hospitals in the region.^2^ This yielded a sample size of 196. Because the study was to be done at four levels of service delivery (HC-II, -III, -IV and General Hospital), the sample size was adjusted to cater for clustering. Assuming the clusters were of equal weights, the average number of responses expected per cluster, b, was (196/4) = 49.

The Design Effect (DE), was then calculated using the formula, DE = 1+ (b-1) roh where roh was the expected inter-cluster variability estimated at 0.20.

DE = 1+ (49-1)0.2 =10.6

This gave a sample size, N=196*10.6 =2,078. After an adjustment of 10% for missing data and incomplete records, this came to a final sample size of 2,309.

**Supplement to Figure 2**

“Other” antibiotics (frequency of prescriptions)

- Other Access antibiotics: Tetracycline (24), Cefalexin (13), Amoxicillin/Clavulanic acid (5), Cloxacillin (3), Nitrofurantoin (3), and Doxycycline (3)
- Other Watch antibiotics: Azithromycin (21), Ciprofloxacin (11), Cefixime (3), and Cefotaxime (1)
- Other unclassified antibiotics: Ceftriaxone/sulbactam (7), Amoxicillin/Flucloxacillin (1), Tinidazole (1)

**References**

1. Kish L. *Survey Sampling*. New York, London: John Wiley & Sons, Inc., 1965.

2. Gwimile JJ, Shekalaghe SA, Kapanda GN *et al.* Antibiotic prescribing practice in management of cough and/or diarrhoea in Moshi Municipality, Northern Tanzania: cross-sectional descriptive study. *Pan Afr Med J* 2012; **12**: 103-.

**Health Facility Tool**

| **Health Facility Tool - Antimicrobial Consumption and Use Surveillance** | |
| --- | --- |
| Z1. Unique ID |  |
| Z2. Enumerator____________________________________ | Z3. Name of Health Facility  __________________________________ |
| Z4. Date(s) of data collection:___________________ | Time: Start_____________  End ______________  Z5. Time: ……… in complete minutes |
| **A. HEALTH FACILITY CHARACTERISTICS** | |
| A1. Type of health facility  [1] Public  [2] Private-Not-For-Profit  [3] Other (specify)  A1a Location  [1] Urban  [2] Rural | A2. Health services offered  [1] Out-patient department  [2] Pediatrics and Child Health  [3] Obstetrics and Gynecology  [4] Medicine  [5] General surgery  [6] Sexual and Reproductive Health  [7] In-patient services  [8] Antenatal services  [9] Laboratory services  [10] Microbiological laboratory services  [11] Other (Specify) |
| A3. Level of health facility  [1] General Hospital  [2] Health Center level IV (Four)  [3] Health Center level III (Three)  [4] Health Center level II (Two)  [5] Regional Referral Hospital | A4. OPD attendance day preceding survey |
| A5. Total OPD attendance in five working (Mon to Fri, excluding public holidays) days preceding survey day | A6. Bed capacity  A6a Number of patients admitted to health facility on day preceding survey  A6b Total admissions in the five working (Mon to Fri, excluding public holidays) days preceding survey day |
| A7. Total number of staff at health facility  A7a. Number of healthcare workers on duty on the day of survey | A8. Number by cadre (only health workers)  A8a. Specialist/Consultant ________  A8b. Medical doctors _____  A8c. Clinical Officers_____  A8d. Pharmacists______  A8e. Pharmacy technician (Dispensers)______  A8f. Nurses and midwives_____  A8g. Laboratory personnel_____  A8h. Records personnel____  A8i. Anesthetists____  A8j. Dental officers_____  A8k. Orthopedic officers_____  A8l. Intern medical doctors_____  A8m. Intern pharmacists _____  A8n. Intern nurses or midwives______  A8o. Other interns (specify)______  A8p. Nursing Assistant ______  A8q. Health assistant______  A8r. Other (specify)_____ |
| A9. Presence of the following at Health facility   \|  \| 1. Yes \| 1. No \| \| --- \| --- \| --- \| \| 1. Uganda Clinical Guidelines \|  \|  \| \| 1. Training on the treatment guidelines for common infections in the last 6 months \|  \|  \| \| 1. Essential Medicines and Health Supplies List of Uganda \|  \|  \| \| 1. IPC Information Education and Communication materials \|  \|  \| \| 1. Other (specify) \|  \|  \| | A10. Have the Medicine Management Supervisors visited this health facility in the last 2 months?  [1] Yes  [2] No |
| A11. Implementing partners supporting health facility  [1] Yes  [2] No | A12. State implementing partner  A12a. Area supported by the implementing partner |
| A13. Does the health facility have the following committees?  *Tick all that apply*  A13a.Medicines and Therapeutic Committee  A13b. Antimicrobial Stewardship (sub) Committee  A13c. Infection Prevention and Control (sub) Committee  A13d. Pharmacovigilance committee | A14. State date when they last held a meeting?  A13a.Medicines and Therapeutic Committee ____________  A13b. Antimicrobial Stewardship Committee ____________  A13c. Infection Prevention and Control Committee_________  A13d. Pharmacovigilance committee_____________________ |
| A15. Has the health facility performed the following in the last two months? ?   \|  \| [1] Yes  [2] No \| \| --- \| --- \| \| 1. Audit of antimicrobial consumption and use and feedback \|  \| \| 1. Assessment of Hand Hygiene compliance \|  \| \| 1. Monitored the performance of microbiology laboratory if present \|  \| |  |
| A16. Do you have a microbiology laboratory to support the diagnosis of the most common infections at your health facility?  [1] Yes  [2] No | A17. If health facility has microbiology lab, does you conduct the following tests at this health facility?   \| B15. Diagnostic tests available \| Available at health facility  [1] Yes  [2] No \| \| --- \| --- \| \| 1. Hepatitis B test \|  \| \| 1. HIV rapid test \|  \| \| 1. Malaria Rapid diagnostic test* \|  \| \| 1. Malaria microscopy \|  \| \| 1. Pregnancy test \|  \| \| 1. Syphilis test \|  \| \| 1. Differential count \|  \| \| 1. Hemoglobin test \|  \| \| 1. Urine dipstick \|  \| \| 1. Urinalysis \|  \| \| 1. Typhoid test \|  \| \| 1. TB LAM rapid test \|  \| \| 1. COVID19 rapid test \|  \| \| 1. COVID19 PCR test \|  \| \| 1. Fullblood count* \|  \| \| 1. Gram stain \|  \| \| 1. High vaginal swab analysis \|  \| \| 1. Wound swab analysis \|  \| \| 1. Urethral and rectal swab analysis \|  \| \| 1. Nasopharyngeal and oropharyngeal swab analysis \|  \| \| 1. CSF analysis \|  \| \| 1. Gene Xpert \|  \| \| 1. Toxo IgG/IgM \|  \| \| 1. CMV IgG/IgM \|  \| \| 1. Microbiology GGT \|  \| \| 1. Bacterial identification tests \|  \| \| 1. Bacteria susceptibility testing \|  \| \| 1. Helicobacter pylori IgG/IgM \|  \| \| 1. Blood culture \|  \| \| 1. Chemistry analyzer \|  \| \| 1. Stool analysis \|  \| |

**OPD REGISTER ABSTRACTION TOOL FOR UNDER-FIVE CHILDREN**

|  | |
| --- | --- |
| 1. **HEALTH FACILITY CHARACTERISTICS** | |
| A1. Unique ID | A2. Enumerator initials____________________________________ |
| A3. Date of data collection:___________________ (dd-mm-yyyy) |  |
| A4. Name of Health Facility ***(selected health facilities provided on last page)*** ________________________________ | A5. Health facility type   1. Government health facility 2. Private not for profit 3. Other |
| A6. Health facility level   1. Health Centre, level II 2. Health Centre, level III 3. Health Centre, level IV 4. General hospital 5. Regional Referral Hospital 6. Other | A7. District of health facility   1. Bududa 2. Bulambuli 3. Jinja 4. Manafwa 5. Mbale 6. Namisindwa 7. Sironko 8. Other |
| A8. Health sub-district ______________________________ |  |
| **B. UNDER-FIVE CHILD CASE (100 cases from January 2019 to December 2020)** | |
| Time: Start_____________  End ______________  B1. Time: ……… in complete minutes | B2. Consultation date (*Date the child visited the health facility*)  __________________________(dd-mm-yyyy) |
| B3. Serial number present  [1] Yes  [2] No | B4. Quote the serial number  ___________________ |
| B5. Name of patient stated  [1] Yes  [2] No | B6. Initials of patient  ---------------------------------- |
| B7. Residence stated  [1] Yes  [2] No | B7a Village____________________  B7b Parish ____________________  B7c Sub-county or county________________________  B7d District _____________________ |
| B8. How old was the child? (in complete months)_________ (if age not recorded, 999) | B9. Gender  [1] Male  [2] Female |
| B10. Weight of child in kilograms ______________ (if weight is not recorded, 999) | B11. Next of kin stated  [1] Yes  [2] No |
| B12. Classification of attendance  [1] New attendance  [2] Re-attendance |  |
| B13. What diagnosis(es) does the case present with for that visit  (*Tick where appropriate-multiple answers allowed*)   \|  \| 1. Yes 2. No \| \| --- \| --- \| \| 1. Malaria \|  \| \| 1. Pneumonia (non-severe) \|  \| \| 1. Severe pneumonia \|  \| \| 1. Cough or cold (acute upper respiratory tract infection) \|  \| \| 1. Diarrhea- acute [watery] \|  \| \| 1. Cholera \|  \| \| 1. Dysentery \|  \| \| 1. Diarrhea-persistent (>14 days) \|  \| \| 1. Measles \|  \| \| 1. Otitis media acute and chronic (ear infection) \|  \| \| 1. Bacterial conjunctivitis \|  \| \| 1. Urinary tract infection \|  \| \| 1. Tonsillitis \|  \| \| 1. Skin infections \|  \| \| 1. Undifferentiated fever (fever with no known cause) \|  \| \| 1. Other (specify in next question) \|  \| | B14. Specified diagnosis  (if other in previous question):_______________  Any diagnostic tests done on the patient?  [1] Yes  [2] No  List the diagnostic tests done on the patient…  Only diagnostic testing for malaria and tuberculosis done?  [1] Yes  [2] No |
| \| B15. Diagnostic tests available \| Available at health facility  [1] Yes  [2] No \| \| --- \| --- \| \| 1. Hepatitis B test \|  \| \| 1. HIV rapid test \|  \| \| 1. Malaria Rapid diagnostic test* \|  \| \| 1. Malaria microscopy \|  \| \| 1. Pregnancy test \|  \| \| 1. Syphilis test \|  \| \| 1. Differential count \|  \| \| 1. Hemoglobin test \|  \| \| 1. Urine dipstick \|  \| \| 1. Urinalysis \|  \| \| 1. Typhoid test \|  \| \| 1. TB LAM rapid test \|  \| \| 1. COVID19 rapid test \|  \| \| 1. COVID19 PCR test \|  \| \| 1. Fullblood count* \|  \| \| 1. Gram stain \|  \| \| 1. High vaginal swab analysis \|  \| \| 1. Wound swab analysis \|  \| \| 1. Urethral and rectal swab analysis \|  \| \| 1. Nasopharyngeal and oropharyngeal swab analysis \|  \| \| 1. CSF analysis \|  \| \| 1. Gene Xpert \|  \| \| 1. Toxo IgG/IgM \|  \| \| 1. CMV IgG/IgM \|  \| \| 1. Microbiology GGT \|  \| \| 1. Bacterial identification tests \|  \| \| 1. Bacteria susceptibility testing \|  \| \| 1. Helicobacter pylori IgG/IgM \|  \| \| 1. Blood culture \|  \| \| 1. Chemistry analyzer \|  \| \| 1. Stool analysis \|  \| | |
| **C. DRUGS AND TREATMENT** | |
| C1. Drug name indicated  [1] Yes  [2] No |  |
| \| C2. Name of drug \| I  Strength or concentration e.g. 250mg or 125mg/ml \| II  Number or quantity of units per dose (***indicate the units*** e.g. mg, mls) \| III  Number of doses per day \| IV  Number of days the drug is to be taken \| \| --- \| --- \| --- \| --- \| --- \| \| 1. Amoxicillin \|  \|  \|  \|  \| \| 1. Amoxicillin/Clavulanic acid \|  \|  \|  \|  \| \| 1. Ampicillin \|  \|  \|  \|  \| \| 1. Azithromycin \|  \|  \|  \|  \| \| 1. Cefalexin \|  \|  \|  \|  \| \| 1. Cefixime \|  \|  \|  \|  \| \| 1. Cefotaxime \|  \|  \|  \|  \| \| 1. Ceftriaxone \|  \|  \|  \|  \| \| 1. Chloramphenicol \|  \|  \|  \|  \| \| 1. Ciprofloxacin \|  \|  \|  \|  \| \| 1. Clarithromycin \|  \|  \|  \|  \| \| 1. Clindamycin \|  \|  \|  \|  \| \| 1. Cloxacillin \|  \|  \|  \|  \| \| 1. Co-trimoxazole \|  \|  \|  \|  \| \| 1. Doxycycline \|  \|  \|  \|  \| \| 1. Erythromycin \|  \|  \|  \|  \| \| 1. Gentamycin \|  \|  \|  \|  \| \| 1. Kanamycin \|  \|  \|  \|  \| \| 1. Metronidazole \|  \|  \|  \|  \| \| 1. Penicillin \|  \|  \|  \|  \| \| 1. Streptomycin \|  \|  \|  \|  \| \| 1. Tetracycline \|  \|  \|  \|  \| \| 1. Artemether/Lumefantrine \|  \|  \|  \|  \| \| 1. Artesunate injection \|  \|  \|  \|  \| \| 1. Artesunate suppositories \|  \|  \|  \|  \| \| 1. Quinine tabs \|  \|  \|  \|  \| \| 1. Quinine syrup \|  \|  \|  \|  \| \| 1. Quinine injection \|  \|  \|  \|  \| \| 1. Artesunate/Amodiaquine tabs \|  \|  \|  \|  \| \| 1. Artesunate/Amodiaquine syrp \|  \|  \|  \|  \| \| 1. Dihydroartemisin/piperaquine tabs \|  \|  \|  \|  \| \| 1. Zinc tablets \|  \|  \|  \|  \| \| 1. Oral Rehydration Salts (ORS) \|  \|  \|  \|  \| \| 1. ORS and Zinc tablets \|  \|  \|  \|  \| \| 1. Paracetamol tabs \|  \|  \|  \|  \| \| 1. Paracetamol syrup \|  \|  \|  \|  \| \| 1. Paracetamol suppositories \|  \|  \|  \|  \| \| 1. Ibuprofen tabs \|  \|  \|  \|  \| \| 1. Ibuprofen suppositories \|  \|  \|  \|  \| \| 1. Diclofenac tabs \|  \|  \|  \|  \| \| 1. Diclofenac injection \|  \|  \|  \|  \| \| 1. Mebendazole tabs \|  \|  \|  \|  \| \| 1. Mebendazole suspension \|  \|  \|  \|  \| \| 1. Albendazole tabs \|  \|  \|  \|  \| \| 1. Albendazole suspension \|  \|  \|  \|  \| \| 1. Levamisole liquid \|  \|  \|  \|  \| \| 1. Other1 (Specify) \|  \|  \|  \|  \| \| 1. Other2 (Specify) \|  \|  \|  \|  \| \| 1. Other3 (Specify) \|  \|  \|  \|  \| \| 1. Other4 (Specify) \|  \|  \|  \|  \| \| 1. Other5 (Specify) \|  \|  \|  \|  \| | |
| C3. Type or nature of patient visit  [1] Index visit  [2] Referral to this health facility |  |
| C4. “Referral IN” number indicated  [1] Yes  [2] No  [3] Not Applicable | C5. “Referral IN” number |
| C6. “Referral OUT” number indicated  [1] Yes  [2] No  [3] Not Applicable | C7. “Referral OUT” number |
